# Cryosurgery as an Option for the Treatment of Vascular Lesions of the Oral Cavity

**DOI:** 10.1155/2017/8529016

**Published:** 2017-08-02

**Authors:** Pedro Thalles Bernardo de Carvalho Nogueira, Mariana Maria Castro Jatobá Remigio, Andreza Maria Correia de Queiroz, Andréia Aparecida da Silva, José Rodrigues Laureano Filho

**Affiliations:** ^1^Universidade Tiradentes (UNIT), Maceió, AL, Brazil; ^2^Oral and Maxillofacial Surgery, Hospital Universitário Oswaldo Cruz (HUOC), School of Dentistry, Universidade de Pernambuco (UPE), Recife, PE, Brazil; ^3^Oral and Maxillofacial Surgery, Universidade do Sagrado Coração (USC), Bauru, SP, Brazil; ^4^Dentistry, UNIT, Maceió, AL, Brazil; ^5^Doctoral Program in Oral and Maxillofacial Surgery, USC, Bauru, SP, Brazil; ^6^Oral and Maxillofacial Surgery, Pernambuco School of Dentistry (FOP), Universidade de Pernambuco (UPE), Camaragibe, PE, Brazil

## Abstract

Cryosurgery is a treatment modality consisting in the destruction of tissue by the application of extremely low temperatures. This causes irreversible damage to cellular metabolism, leading to tissue destruction within minutes, a mechanism that may be beneficial when used in diseased tissues. Because cryosurgery is effective, simple, and easy to perform, it has been used in the treatment of lesions in both medical and dental fields. This technique provides many advantages, such as easy operation, absence of intraoperative bleeding, and low infection rate. We report the case of a patient with a hemangiomatous lesion of the oral cavity who was treated with liquid nitrogen spray cryosurgery, with successful results at 18-month follow-up.

## 1. Introduction

Cryotherapy or cryosurgery is a form of therapy consisting in the in situ destruction of tissue by the application of extremely low temperatures [1, 2]. The resulting cryogenic lesion is characterized by sharply circumscribed necrosis that corresponds to the volume of previously frozen tissue, and this term is used to refer to the effect of tissue freezing [3].

Although reports of the use of ice for therapeutic purposes date back to about 3000 BC [4, 5], it was only in the mid-1960s that the basic features of cryosurgical technique, that is, rapid freezing, slow thawing, and repetition of the freeze-thaw cycle, were established [6]. Since then, this treatment modality has been successfully applied across a wide range of medical fields, such as dermatology, proctology, gynecology, neurosurgery, ophthalmology, general surgery, and head and neck surgery [4, 7].

In oral and maxillofacial surgery, cryotherapy is the gold standard for the treatment of various types of lesions occurring in the oral cavity. It can be used as a single treatment for mucocele [8], trigeminal neuralgia [9], leukoplakia, hemangioma, pyogenic granuloma, HPV lesions, actinic cheilitis, lichen planus, and fibromas [2, 10, 11] and as a complementary treatment in cases of bone lesions with high recurrence rates, such as ameloblastomas, myxomas, keratocystic odontogenic tumor, ossifying fibroma, and central giant cell lesions. The standard treatment for these lesions is usually aggressive and involves resection with safety margins, which often results in major cosmetic and functional defects for the patient. In this respect, cryosurgery provides the most effective alternative to ensure the safety margin without causing major defects, since it destroys deep diseased tissue through in situ freezing [11].

Given the wide applicability of cryosurgery in the field of oral and maxillofacial surgery, we report a case of hemangioma of the oral cavity successfully treated with cryosurgery.

## 2. Case Report

A 65-year-old white woman presented to the Department of Oral and Maxillofacial Surgery at the Pernambuco School of Dentistry, Universidade de Pernambuco, Northeastern Brazil, complaining of a purplish lesion on the left lateral border of the tongue. The patient reported that the onset of the lesion had occurred about 2 years earlier and that it continued to grow slowly and asymptomatically. The patient had diabetes and hypertension. She had no family history of malignancies and no history of smoking or alcohol consumption.

**Figure 1:**
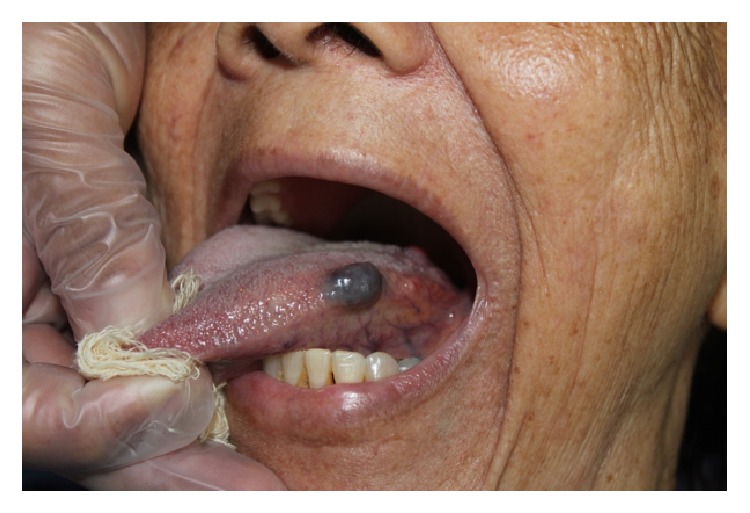
Hemangiomatous lesion on the left lateral border of the tongue.

**Figure 2:**
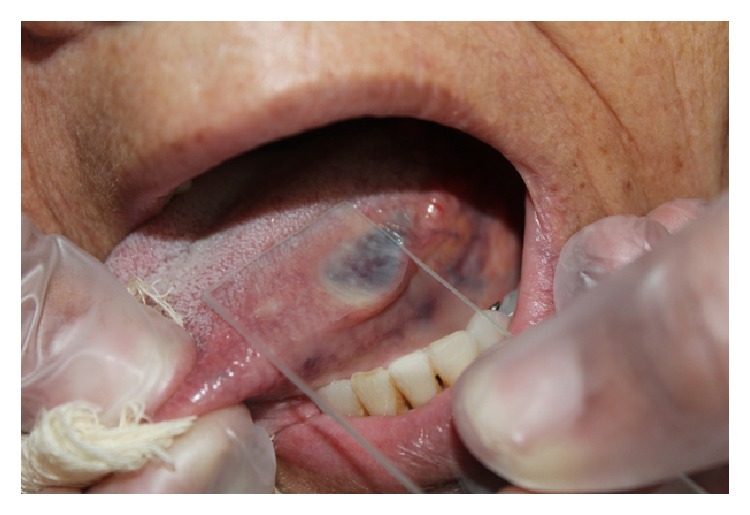
Diascopy (pressure on the lesion with a glass slide) showing color change.

Extraoral physical examination revealed no changes in the patient's facial anatomy. Cervical lymph node characteristics were within the normal range. Intraoral examination revealed that the patient was completely edentulous in the maxilla and partially edentulous in the mandible. An asymptomatic, purplish, sessile nodular lesion, firm to palpation, and measuring about 1.5 cm in its greatest diameter was found on the left lateral border of the tongue (Figure 1). Diascopy, performed by applying pressure on the lesion with a glass slide, showed that the lesion had an ischemic pattern. Fine-needle aspiration was performed and the result was positive for bloody content, suggestive of a hemangiomatous lesion (Figure 2).

Based on clinical findings, the diagnosis of hemangioma was made. It is worth noting that, in cases of hemangiomatous lesions, incisional biopsy is totally contraindicated, because even partial manipulation of the lesion may cause significant bleeding.

**Figure 3:**
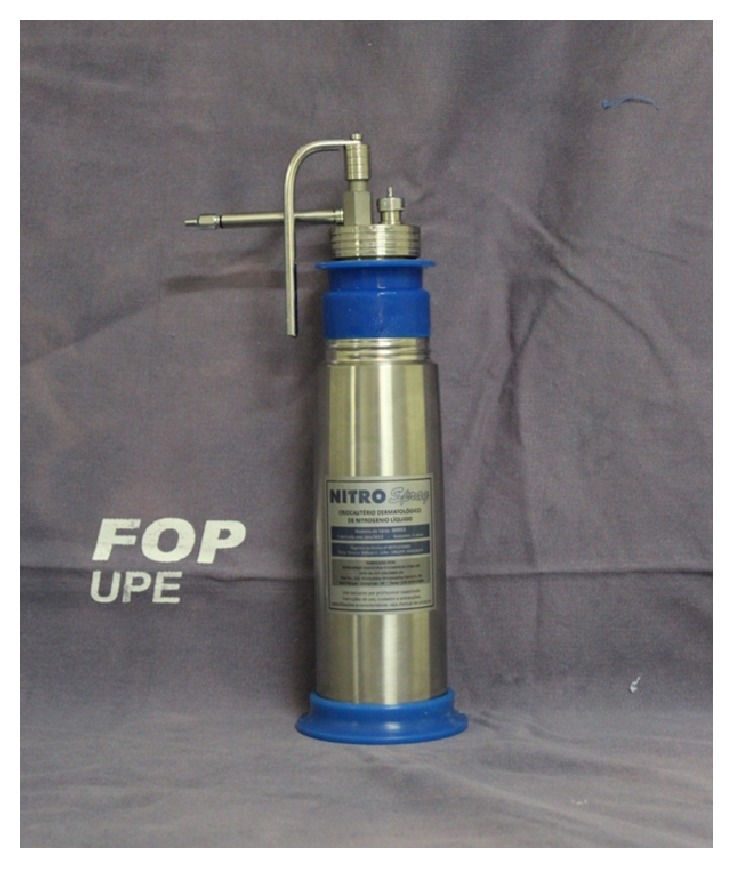
Cryocautery system: liquid nitrogen container and pump system used in the present case.

Several treatment modalities reported in the literature for this condition were reviewed and, for this case, cryosurgery with liquid nitrogen was chosen as definitive treatment (Figure 3).

**Figure 4:**
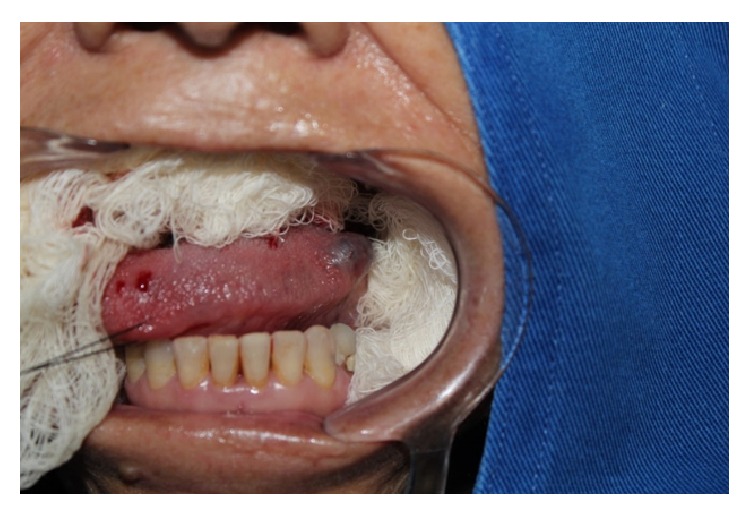
Tongue pulled to the right and adjacent tissues protected by gauze with Vaseline.

The patient was hemodynamically stable and cryosurgery was performed under local anesthesia in an outpatient setting. The surgical site was disinfected with 2% chlorhexidine digluconate gel, followed by anesthesia of the tongue apex and of the affected area by anesthetic block of the lesion site with lidocaine with epinephrine (1 : 100,000). The tongue apex was sutured with 3-0 nylon, the tongue was pulled to the right, and adjacent tissues (tongue, cheek mucosa, palate, and teeth) were protected by gauze with Vaseline (Figure 4).

**Figure 5:**
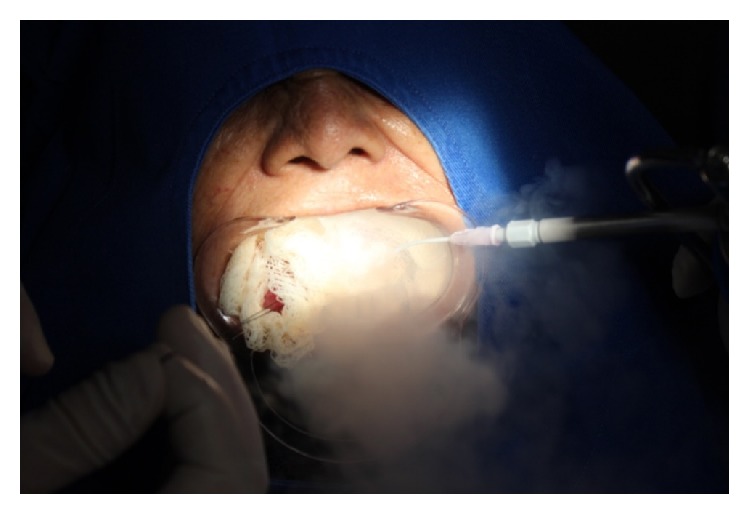
Application of liquid nitrogen on the lesion.

**Figure 6:**
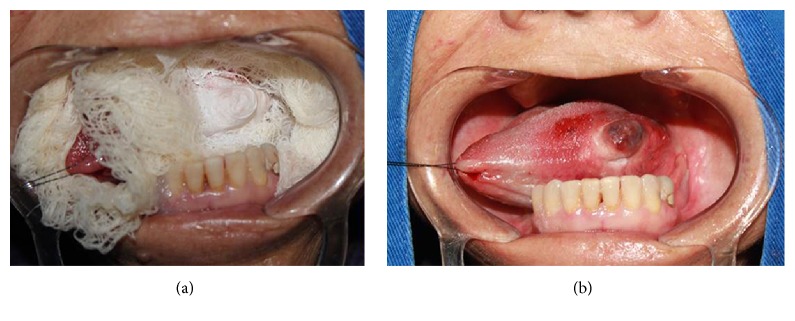
(a) Appearance of the frozen lesion and (b) formation of perilesional edema and erythema in the immediate postoperative period.

Liquid nitrogen spray cryosurgery was performed in a single session of two cycles of 1 minute each, with a 2-minute interval between cycles (Figure 5). Immediately after the procedure, the lesion was whitish in color, characterizing the freezing process, and, a few minutes later, after thawing, edema and erythema formations were observed in the treated area (Figure 6).

**Figure 7:**
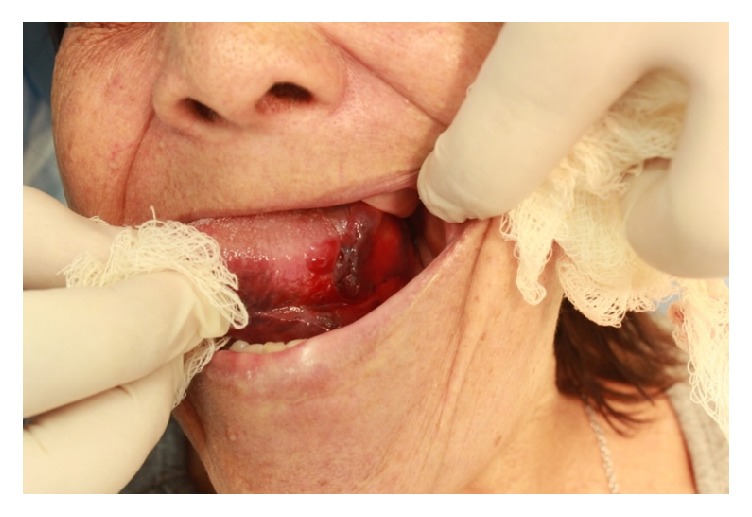
Postoperative week 1.

**Figure 8:**
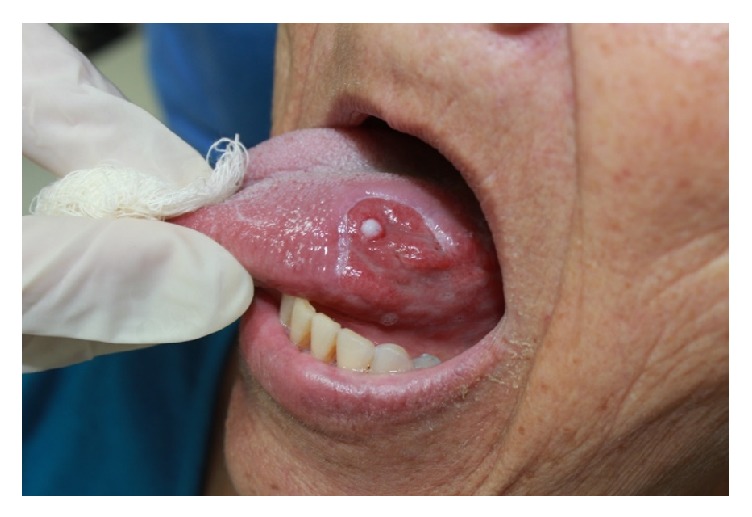
Postoperative week 3.

**Figure 9:**
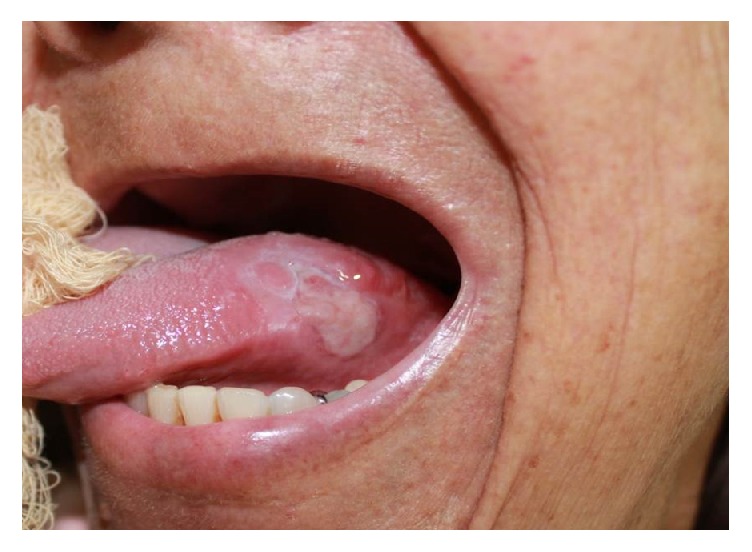
Postoperative week 6.

**Figure 10:**
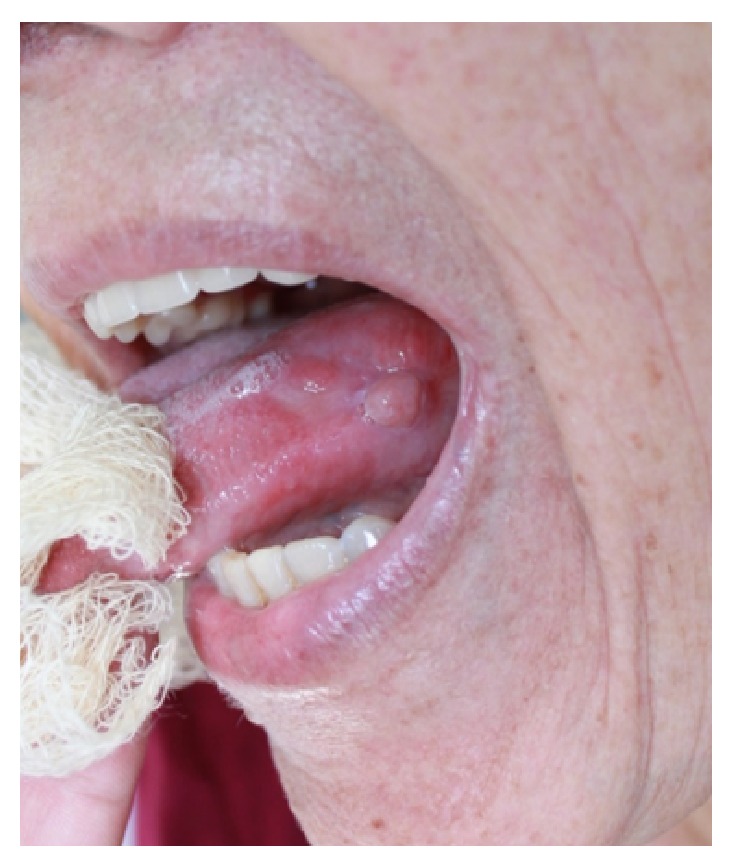
Postoperative week 8.

The patient returned for outpatient follow-up at 1, 3, 6, and 8 weeks postoperatively (Figures 7, 8, 9, and 10). On the first postoperative days, the patient complained of mild pain, which was managed with dipyrone (500 mg) every 6 hours for 5 days. There were no signs of infection during the healing period.

**Figure 11:**
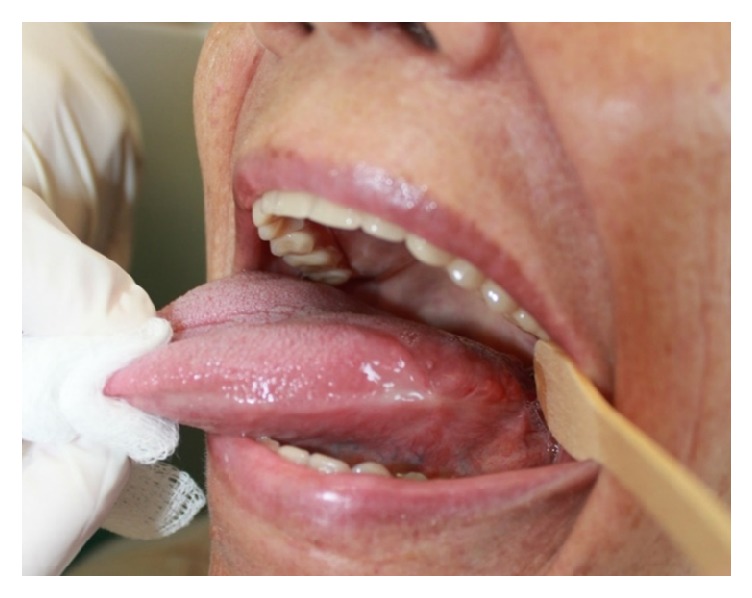
Postoperative month 18.

At the time of the last follow-up, 18 months after treatment, the patient was clinically well, without sensory changes in the tongue, abnormal speech, or impaired swallowing and with no signs of recurrence of the lesion. A slight scar retraction was observed at the lesion site (Figure 11).

## 3. Discussion

Cryosurgery consists in freezing the tissues in order to obtain local destructive effects and subsequent therapeutic action [1]. Over time, several authors have reported on the successful use of this modality in the treatment of various types of lesions. Yeh [2] and Rubinsky and Onik [4] described the use of cryosurgery in several areas, such as dermatology, oncology, hepatology, proctology, urology, ophthalmology, general surgery, and oral and maxillofacial surgery.

Hausamen [12], for 3 years, successfully treated several diseases of the oral cavity, including hemangioma, leukoplakia, and squamous cell carcinoma. In agreement with the results obtained and reported in the literature [2, 13–15], in the case reported here, cryosurgery proved to be a simple, fast, and extremely effective procedure in the treatment of hemangioma of the oral cavity.

Although there are no studies comparing efficacy among the various cryosurgical techniques, application of liquid nitrogen with a cotton swab has proven to be practical and effective in the treatment of several oral lesions [2, 8, 15]. However, in the present study, due to the size of the lesion, we believed that a deeper cryonecrosis would be necessary and, therefore, we chose to use a liquid nitrogen spray technique, as reported in the study conducted by Hartmann et al. [13].

In agreement with Graham and Barham [5] and Gallardo et al. (2000) [16], although a number of cryogenic agents are available in the market, currently, liquid nitrogen should be the agent of choice because it reaches the lowest temperatures (approximately 196°C) and is a relatively inexpensive and easy-to-handle substance. As observed in the case reported here, treatment of oral lesions using cryosurgery is well accepted by patients and the procedure is well tolerated under local anesthesia, as also reported by Li [17].

Cryotherapy is considered simple, fast, and easy to perform, and the formation of edema and erythema in the area exposed to the cryogenic agent is evident immediately after the procedure. There are no reports of postoperative infection, difficulty breathing, or allergic reactions to the cryogenic agent. All studies have reported the presence of mild pain, which is managed solely with the use of peripherally acting analgesics [12, 15, 18, 19].

## 4. Final Considerations

Based on data reported in the literature and obtained in the present case report, cryosurgery is a safe and extremely effective procedure in the treatment of lesions occurring in the oral and maxillofacial region. Nevertheless, despite the scientifically proven efficacy and routine application of cryosurgery across many medical specialties, this treatment modality is still rarely used in dentistry.
